# Fetal Adrenal Mass on Ultrasound: A Rare Prenatal Challenge

**DOI:** 10.7759/cureus.105650

**Published:** 2026-03-22

**Authors:** Nalini Arora, Gaurav Khastgir, Dipankar Saren

**Affiliations:** 1 Obstetrics and Gynaecology, Employees’ State Insurance-Post Graduate Institute of Medical Sciences and Research (ESI-PGIMSR), Employees’ State Insurance Corporation (ESIC) Medical College Hospital and Occupational Disease Centre, Kolkata, IND

**Keywords:** fetal neuroblastoma, neonatal neuroblastoma, obstetrics ultrasonography, prenatal ultrasound diagnosis, suprarenal mass

## Abstract

Congenital neuroblastoma is a rare fetal malignancy arising from neural crest cells, most commonly from the adrenal gland. We report a case of a primigravida in whom a left suprarenal mass was incidentally detected on routine third-trimester ultrasonography at 35 weeks’ gestation in an uncomplicated pregnancy, and conservative antenatal management with close fetal surveillance with multidisciplinary care was adopted. The patient delivered a live male neonate at term by cesarean section for failed induction. Postnatal computed tomography demonstrated a solid left adrenal mass with internal calcifications and retroperitoneal extension, without evidence of metastasis. The diagnosis of neuroblastoma was confirmed by ultrasonography-guided fine-needle aspiration cytology. The infant was treated with combination chemotherapy and tolerated the treatment uneventfully and remained well on follow-up. This case highlights the value of antenatal diagnosis, which facilitates early surveillance and management through a multidisciplinary approach, thereby optimizing outcomes in congenital neuroblastoma.

## Introduction

Neuroblastic tumors, such as neuroblastomas, ganglioneuroblastomas, and ganglioneuromas, constitute a spectrum of poorly differentiated tumors that arise from embryonic neural crest cells. Congenital neuroblastoma is the second most common neonatal tumor, accounting for 20% of all congenital tumors [1]. Its incidence ranges from 1 in 10,000 to 1 in 100,000 live births [2]. Advances in ultrasonography (USG) and prenatal diagnosis enable more accurate detection of fetal tumors. We report the case of a primigravida with a fetal suprarenal mass detected prenatally on third-trimester USG. This case highlights that an early diagnosis offers the potential for prompt neonatal treatment and better survival chances, especially in tumors such as neuroblastoma, due to the inverse relationship between age and survival.

## Case presentation

**Figure 1:**
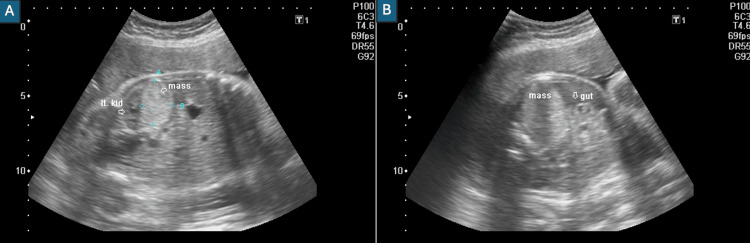
Prenatal ultrasonography demonstrating the echogenic lobulated space-occupying lesion in the fetal suprarenal region. (A) Echogenic mass displacing the left kidney. (B) Relationship of the mass with the fetal gut.

A 20-year-old primigravida with no comorbidities underwent USG at 35 weeks’ gestation, which demonstrated a 5 × 4.2 cm lobulated lesion in the left suprarenal region displacing the left kidney, with some lobules showing vascularity on colour Doppler (Figures 1A, 1B).

No other structural anomalies were noted, and the liquor volume and estimated fetal weight were appropriate for gestational age. The patient was admitted for further investigations and inpatient management. After discussion with the pediatrics and neonatology teams, a decision was made to proceed with conservative management. She underwent an emergency cesarean section at 38 weeks of gestation, subsequent to failed induction of labor, and delivered a liveborn baby boy weighing 2.3 kg with no gross congenital malformations. The neonate underwent a non-contrast-enhanced computed tomography (NCCT) of the abdomen on the second day of life.

Imaging revealed a large, solid left adrenal mass with internal calcific foci, infiltrating the retroperitoneum, encasing the abdominal aorta, and displacing the left kidney posterolaterally. The spinal cord or neural foramina were not involved, and there was no radiologic evidence of metastasis. The findings were suggestive of a stage L2 neuroblastoma (International Neuroblastoma Risk Group Staging System [3]), which was confirmed by USG-guided fine-needle aspiration cytology at the second month of life. The infant received chemotherapy with cyclophosphamide, cisplatin, and Adriamycin and currently remains asymptomatic.

## Discussion

Neuroblastomas are the most common prenatally diagnosed thoracoabdominal tumors and account for 50% of neonatal malignancies [4]. They are slightly more common in boys, with a male-to-female sex ratio of 1.2:1 [5].

The cause of neuroblastoma is unknown in most cases. Most occur sporadically; around 1-2% are familial and associated with multiple primary tumors, usually occurring at <18 months of age. Germline mutations in the *ALK* and *PHOX2B* genes have recently been linked to the development of hereditary neuroblastomas [6]. Advances in genetics, using single-nucleotide polymorphism (SNP)-based microarray analysis, have identified multiple genetic loci associated with an increased risk of sporadic neuroblastoma [1]. Copy-number variations at 1q21 have also been implicated in pathogenesis, with N-myc amplification linked to tumor aggressiveness [6]. Some authors have also reported cases involving mothers receiving phenobarbitone and phenytoin antenatally, but the association is likely coincidental [1].

Fetal neuroblastoma is usually discovered incidentally during routine obstetric USG. These are most commonly identified in the third trimester, with the earliest gestational age at prenatal diagnosis reported as 23 weeks [7]. The majority of tumors are found in the suprarenal region. These are well-encapsulated tumors that displace the kidneys inferolaterally and demonstrate three distinct sonological morphologies. The appearance varies from a solid, isoechoic mass (50%) to purely hypoechoic cystic and mixed echogenicity. While solid masses usually increase in size, other morphologies have been shown to have a higher propensity for spontaneous resolution [1]. The differential diagnosis of a suprarenal mass includes renal cysts, adrenal hemorrhage, adrenal abscess, subdiaphragmatic extra-lobar pulmonary sequestration, enteric duplication cysts, and cystic Wilms’ tumor [8]. It is crucial to differentiate a cystic neuroblastoma from adrenal hemorrhage, which is the most common cause of an adrenal mass in a neonate, with a prevalence of 2 per 1,000 live births. Adrenal hemorrhages are mostly echogenic and do not show vascularity on color Doppler [9,10].

Fetal neuroblastomas exhibit a clinical behavior ranging from an aggressive metastatic disease to spontaneous regression in utero or shortly after birth. These tumors may metastasize to the fetal liver and placenta and might cause fetal heart failure, resulting in fetal hydrops and polyhydramnios due to catecholamine release. The prognosis is generally excellent in both the early and advanced stages, with a very low risk of recurrence [11].

Though these prenatally diagnosed tumors have been reported to be associated with improved perinatal survival of more than 90% [1], they remain a potential source of morbidity and perinatal mortality, making prenatal counselling crucial and challenging. Maternal complications are rare, but palpitations, flushing, sweating, hypertension, and maternal mirror syndrome have been reported [12]. Therefore, detailed counselling and close maternal and fetal surveillance with serial fourth-weekly USG (as suggested by the Foetal Medicine Foundation) to monitor for tumor growth, placental thickness, fetal hepatomegaly, and other signs of fetal compromise is recommended.

In the index case, the first evidence of an intra-abdominal space-occupying lesion was noted only in the late third trimester. A conservative management approach was agreed upon by the obstetric and pediatric teams, with close antenatal monitoring. Given the radiological extent of the disease noted on the NCCT, the infant was initiated on chemotherapy following histopathologic confirmation of a neuroblastoma.

USG is an accepted modality for screening, detection, and surveillance of these tumors. However, fetal magnetic resonance imaging is indicated to detect fetal metastasis and potential intraspinal extension. Vaginal delivery is aimed at 38 completed weeks of gestation. Neonatal treatment includes medical care with chemotherapy and surgery. Good survival rates are reported even in metastatic tumors with very low recurrence risk [1].

## Conclusions

Fetal adrenal neuroblastoma is the most common extracranial childhood solid tumor with good postnatal prognosis and survival rates. Advances in prenatal diagnosis have enabled early recognition and management. Close antenatal follow-up is crucial for early detection of prenatal complications such as fetal hydrops, polyhydramnios, and preeclampsia. A routine third-trimester USG can facilitate appropriate maternal and fetal surveillance, with the aim of initiating early neonatal treatment and improving survival rates. A collaborative approach with a multidisciplinary team of obstetricians, neonatologists, radiologists, and pediatric oncologists, involving the patient and her family in shared decision-making, is essential to optimize outcomes.
